# Early exposure to UV radiation overshadowed by precipitation and litter quality as drivers of decomposition in the northern Chihuahuan Desert

**DOI:** 10.1371/journal.pone.0210470

**Published:** 2019-02-04

**Authors:** Daniel B. Hewins, Hanna Lee, Paul W. Barnes, Nathan G. McDowell, William T. Pockman, Thom Rahn, Heather L. Throop

**Affiliations:** 1 Biology Department, Rhode Island College, Providence, Rhode Island, United States of America; 2 NORCE Norwegian Research Centre, Bjerknes Centre for Climate Research, Bergen, Norway; 3 Department of Biological Sciences, Loyola University, New Orleans, Louisiana, United States of America; 4 Pacific Northwest National Laboratory, Richland, Washington, United States of America; 5 Department of Biology, University of New Mexico, Albuquerque, New Mexico, United States of America; 6 Earth and Environmental Sciences Division, Los Alamos National Laboratory, Los Alamos, New Mexico, United States of America; 7 School of Earth and Space Exploration, Arizona State University, Tempe, Arizona, United States of America; 8 School of Life Sciences, Arizona State University, Tempe, Arizona, United States of America; Universidade de Lisboa Instituto Superior Tecnico, PORTUGAL

## Abstract

Dryland ecosystems cover nearly 45% of the Earth’s land area and account for large proportions of terrestrial net primary production and carbon pools. However, predicting rates of plant litter decomposition in these vast ecosystems has proven challenging due to their distinctly dry and often hot climate regimes, and potentially unique physical drivers of decomposition. In this study, we elucidated the role of photopriming, *i.e.* exposure of standing dead leaf litter to solar radiation prior to litter drop that would chemically change litter and enhance biotic decay of fallen litter. We exposed litter substrates to three different UV radiation treatments simulating three-months of UV radiation exposure in southern New Mexico: no light, UVA+UVB+Visible, and UVA+Visible. There were three litter types: mesquite leaflets (*Prosopis glandulosa*, litter with high nitrogen (N) concentration), filter paper (pure cellulose), and basswood (*Tilia* spp, high lignin concentration). We deployed the photoprimed litter in the field within a large scale precipitation manipulation experiment: ∼50% precipitation reduction, ∼150% precipitation addition, and ambient control. Our results revealed the importance of litter substrate, particularly N content, for overall decomposition in drylands, as neither filter paper nor basswood exhibited measurable mass loss over the course of the year-long study, while high N-containing mesquite litter exhibited potential mass loss. We saw no effect of photopriming on subsequent microbial decay. We did observe a precipitation effect on mesquite where the rate of decay was more rapid in ambient and precipitation addition treatments than in the drought treatment. Overall, we found that precipitation and N played a critical role in litter mass loss. In contrast, photopriming had no detected effects on mass loss over the course of our year-long study. These results underpin the importance of biotic-driven decomposition, even in the presence of photopriming, for understanding litter decomposition and biogeochemical cycles in drylands.

## Introduction

Arid and semiarid ecosystems (hereafter ‘drylands’) are an important component of terrestrial biogeochemical cycles, accounting for approximately 45% of land area [1], 30% of net primary production [2], and nearly 20% of the global soil organic carbon (C) pool [3, 4]. The dryland contribution to the global C budget will likely increase as global climate change projections suggest future expansion of dryland area [5]. Despite the global significance of drylands on the Earth system, controls over biogeochemical processes are poorly understood in drylands relative to mesic systems [6, 7]. For example, leaf litter decomposition rates are accurately predicted in mesic systems using models that consider simple climatic factors such as temperature and actual evapotranspiration [8, 9] and litter chemistry such as lignin to nitrogen (N) content [10].

While in dryland systems, these models underpredict decomposition rates, creating a gap between modeled and observed decay rates [6]. Recent studies from drylands highlight the potential importance of abiotic drivers under dry conditions [11–14], while studies in wetter conditions suggest that abiotic drivers may be less important than mechanisms such as precipitation-driven biological activity [15, 16].

Among the abiotic factors considered dominant in dryland litter decomposition, is the photochemical degradation of organic compounds by exposure to solar radiation (hereafter ‘photodegradation’). Results from two meta-analyses suggest that solar UV-B radiation and full solar radiation were responsible for less than 25% of decomposition on average [17, 18]. Among the wavelengths involved in photodegradation, UV-B radiation can breakdown lignin [19–23], an organic compound that is often resistant to microbial decomposition. The effects of UV radiation, UV-B (280-315 nm) and UV-A (315-400 nm), on organic matter decay may be an important, but unaccounted for driver of organic matter decomposition, as a large proportion of drylands are high radiation environments. Moreover, our knowledge in the timing and the duration of exposure to solar radiation is limited, however, duration may be linked with the magnitude of the effect [24].

Recent climate modeling studies suggest that some drylands will experience more prevalent droughts in the future as a consequence of climate change [25–27]. Conversely, certain regions may receive above average precipitation [26]. Increased aridity, suggested by climate models, may reduce biotic decomposition, thus increasing the importance of understanding the role of abiotic drivers that largely operate independently of precipitation and moisture [28]. While in regions where precipitation is expected to increase, we expect enhanced biotic activity including plant growth and litterfall, and microbial activity, which may positively influence both abiotic and biotic decomposition.

It is important to consider the synergistic effects of abiotic and biotic processes to elucidate the specific mechanisms and the rate of dryland litter decomposition [29]. Most photodegradation studies observe the sole effects of high solar radiation exposure on litter mass loss, but litter decay in drylands is more realistically a multi-step process that follows a period of exposure to solar radiation prior to leaf abscission (*i.e.* photopriming or also known as photofacilitation) and the abiotic and biotic decomposition starts once litter falls to the soil [30]. However, there is a growing understanding that photodegradation and microbial decomposition occur simultaneously in dry environments and thus there is a need to understand the combined effects of the two in overall decomposition process, although there is still no consensus on what direction and to what degree photodegradation enhances microbial decomposition [30]. One study measuring the interaction between photodegradation and biological decomposition showed that photodegradation and microbial enzyme activity facilitate decay in dry and wet periods [31]. Another study found that microbial decay occurred at night using moisture found in dew, while photodegradation was important during dry periods [32]. On the other hand, one study showed that photodegradation enhanced microbial decomposition in less arid sites compared to hyper-arid sites [33]. In addition, laboratory incubation study found that small amounts of soil changes in moisture largely regulated the rate of dryland litter mass loss [34]. Results from several recent studies suggest that litter photopriming has the potential to encourage biotic decomposition by degrading recalcitrant compounds, thus making the litter more labile and decomposable by microbes [15, 22, 35, 36]. Therefore, understanding the effects of photopriming, defined as exposure to solar radiation occurring prior to abscission of standing dead leaf material, may explain if photodegradation is important for biotic decomposition in drylands, as microbial decomposition may be enhanced due to pre-decay of lignin and other complex compounds forming litter material [35]. However, in many dryland systems, there is a limited time during which photodegradation of litter can occur at the surface due to the likelihood of litter burial by soil-litter mixing. Soil-litter mixing can limit litter exposure to solar radiation and litter covered by soil is protected from UV radiations, negating further photodegradation [13, 29, 37]. Thus, photodegradation is likely to be most important as photopriming during the period of time between leaf senescence and litter fall [37].

Historically, many studies have only focused on understanding differences in decomposition between dry and mesic systems by quantifying discrepancies between observations and model expectations. In our study, we aim to focus on adequately investigating potentially important synergistic effects between abiotic (*i.e.* solar radiation) and biotic decay in drylands.

To investigate the relationship between solar radiation and biological decomposition, we address the following questions: 1) Does photopriming affect microbial litter decomposition rates?, 2) Is precipitation a strong control of decomposition rates in drylands that overcomes the effects of photodegradation?, 3) What interaction effects occur when UV radiation and precipitation are manipulated during litter decomposition process? To address these questions, we established a litter decomposition experiment within a dryland precipitation manipulation study using litter that differed in pre-experimental solar radiation exposure. The solar radiation exposure was equivalent to 3 months UV-B radiation in the southern Chihuahuan Desert in order to simulate photopriming relevant to field conditions. Decomposition rate was assessed as the mass loss of buried litter in mesh litterbags, negating the possibility for additional photodegradation and putatively making this mass loss the result of microbial activity. We hypothesized that 1) mass loss of buried dryland litter will be enhanced on litter pre-explosed to UV radiation, 2) the litter mass loss rate will be faster with increased precipitation as a result of enhanced microbial activity, and 3) there will be a positive interaction between photopriming and precipitation because photoprimed litter will be made susceptible to microbial decomposition, which will be enhanced by elevated levels of precipitation.

## Materials and methods

### Experimental design

This experiment was designed to represent three natural stages of dryland decomposition: 1) photodegradation of standing dead prior to leaf abscission, 2) litterfall to the ground and mixture with soil, and 3) microbial decomposition [37]. To understand the contributions of abiotic and biotic mechanisms during dryland decomposition, we pre-treated the litter material with three different combinations of solar UV-B radiation (see ‘*Litter preparation and pre-treatment*’ for detailed description). Pre-treated litter was then placed in litterbags and deployed in the field to explore the effects of solar radiation pre-treatment on biotic decomposition under three different field precipitation treatments. Note that no manipulations of solar radiation occurred during the field study; hence, this study explores the role that UV and visible radiation play in modifying litter quality prior to microbial decomposition.

### Litter preparation and pre-treatment

We prepared three different types of litter that differed in chemical composition. Cellulosic filter paper (≥98% cellulose, ≤0.007% ash, Whatman 42, GE Healthcare Inc., Piscataway, NJ, USA) and 1.6 mm thick sheets of basswood (*Tilia* sp.) wood (high lignin content, National Balsa, Ware, MA, USA) were used to mimic cellulose and lignin end members, respectively. High N-containing litter of *Prosopis glandulosa* (honey mesquite) was used as representative litter common in deserts in the southwestern United States. Mesquite leaves were collected immediately before senescence from a natural desert environment on the New Mexico State University campus in Las Cruces, New Mexico, USA. Initial C content was 42.7% ± 0.08, 48.1% ± 0.06, and 45.4% ± 0.13 for cellulose paper, basswood, and mesquite, respectively. Initial N content was 2.5% ± 0.09 for mesquite; while filter paper and basswood did not contain measurable N (n = 30 of each litter type).

Photopriming was carried out in a temperature controlled environmental chamber by laying out a single layer of litter 60 cm below lamps covered with filters to establish treatments. The three photopriming treatments were 1) no light; 2) UV-B, UV-A, and visible light wavelengths (hereafter, +UVB treatment); and 3) UV-A and visible light wavelengths (hereafter, -UVB treatment). Litter was exposed to ∼700 kJ m^-2^ total dosage of UV-B (7.6 kJ m^-2^ d^-1^), which was the equivalent of total UV radiation measured in southern New Mexico for June, July and August in year 2009 (http://nadp.nrel.colostate.edu/UVB/, the USDA UV-B Radiation Monitoring Program at Colorado State University). This exposure time was chosen to simulate the maximum UV-B exposure for mesquite litter that would occur between leaf senescence and litterfall. The exposure period was constant without a day/night cycle, which summed to approximately 4 weeks. The exact exposure length varied ±3 days depending on the conditions of the lamps and the filters in order to achieve the total radiation target of 700 kJ m^-2^ (3 month UV-B equivalent). There was no additional lighting inside the chamber except the treatment lamps to prevent additional short-wave radiation exposure of the litter. Throughout the photopriming treatment, the UV lamp output and chamber temperature were regularly monitored. The UV lamps (40-W UV-B 313 fluorescent bulbs, Q-Panel, Cleveland, OH, USA; unweighted UV-B (280-315 nm) and UV-A (315-390 nm) irradiances = 756 and 694 mW m^-2^, respectively) were covered with UV-transparent film (clear cellulose diacetate, JCS Industries, La Mirada, CA, USA) for the +UVB treatment or UV-absorbing film (UV-B absorbing; clear Mylar film, CPFilms, Fieldale, VA, USA) for -UVB treatment. The films were replaced every 3-4 days as needed to maintain the target mean daily UV-B dosage. The spectral irradiance was measured daily with a double-monochrometer UV/Vis spectroradiometer (Model 754, Optronic Laboratories, Orlando, FL, USA) calibrated for wavelength accuracy (4-W fluorescent lamp at wavelengths = 312.9 and 546.1 nm) and absolute responsiveness (200-W tungsten-halogen lamp traceable to a NIST standard), and weighted according to a generalized plant action spectrum to obtain a measure of biologically effective UV irradiance (UV-BBE = 260 mW m^-2^). The transmission spectra of the cellulose diacetate and polyester plastic film used in this study can be found in [38]. The spectral irradiane of the UV lamps enclosed by these two types of film can be found in [39]. Environmental chamber air temperature was maintained at 30-35°C. As the temperature in the chamber during the UV exposure was higher than room temperature, we kept the ‘no light’ treatment samples inside the chamber to maintain similar environmental conditions, while being contained in heavy paper bags to prevent UV transmission.

Once the target radiation exposure was achieved for each batch of litter, the photopriming treatment was terminated. The filter paper and basswood material were cut to approximately 1 × 1 cm^2^ squares. Mesquite leaflets were removed from the rachis. The litter material from all batches were thoroughly homogenized by the same litter type. Litterbags (10 × 20 cm^2^) were constructed from fiberglass window screen (∼0.9 mm openings; Phifer Wire Products, Tuscaloosa, Alabama, USA). Bags were filled with approximately 2 g filter paper, 3 g mesquite leaflets, or 7 g basswood. The selected litter mass corresponds to the amount of each litter required to cover the litterbag area at a single layer thickness.

### Field experiment

The litterbag study was carried out within the Pinyon-Juniper Rainfall Experiment plots at the Sevilleta National Wildlife Refuge Long-Term Ecological Research (LTER) site (34°23′11″N, 106°31′46″ W). The vegetation at the field site was mixture of *Pinus edulis* (piñon pine) and *Juniperus monosperma*(one seed juniper). A 20-year climate record from a nearby Sevilleta LTER meteorological station (Cerro Montoso #42; http://sev.lternet.edu/) indicate that the mean annual temperature was 12.7°C (mean July maximum: 31.0°C, mean December minimum: -3.3°C) and mean annual precipitation was 362.7 mm yr^-1^. Rainfall was manipulated in three replicated blocks for each of three treatment plots: 1) ∼50% precipitation reduction (hereafter ‘drought’), 2) ∼150% precipitation addition (herafter ‘addition’), and 3) ambient control (hereafter ‘ambient’). Precipitation manipulations were calculated relative to the 30-year long-term average. For more information on the site and rainfall manipulation, see [40, 41]. Three 40 m x 40 m plots of each treatment were randomly located across the site, for total of nine plots. Drought was achieved through the use of plexiglass troughs located 1 m above ground, covering ∼50% of the surface area of the plots. Precipitation addition was achieved by irrigating with sprinkler hoses that applied deionized water approximately monthly during the growing season. The water added was municipal water treated by reverse osmosis to achieve electrical conductivity similar to rain water.

Litterbags were arrayed in the field in precipitation treatment plots in the last week of October in 2010 after the end of the growing season and before winter snow fall. Litterbags were buried under 0.5 cm soil to prevent secondary exposure of solar radiation during microbial decomposition. This litter burial mimics common patterns in dryland ecosystems where low vegetative cover leads to high rates of soil transport and litter burial [29, 42, 43]. The litterbags were collected after 0, 1, 3, 6, and 12 months to estimate the rate of decomposition.

### Laboratory analysis

Upon litterbag collection, we processed the contents by partitioning litter and soil using a 1 mm sieve, followed by gently brushing litter by hand to remove soil particles that were lightly adhered to the litter surface. Cellulose often crumbled during handling, so flakes were collected and carefully added to account for their mass. Processed litter and soil were frozen at -80°C for 48 hours, lyophilized using a Freezone 4L (Labconco Corp., Kansas City, MO, USA), weighed, and ground to a fine powder using an 8000D Mixer Mill (SPEX Certiprep Inc., Metuchen, NJ, USA). Subsamples of ground litter were ashed in a muffle furnace at 550° for 6 hours. Additional subsamples were analyzed for C and N content on an ECS 4010 Elemental Analyzer (Costech Analytical Technologies, Valencia, CA, USA). All litter mass loss and CN data were corrected by ash content and expressed on an ash-free mass basis to exclude mass gain from adhering soil particles.

### Statistical analysis

Decay constants (*k* values) were estimated using a single-pool exponential decay model,

$${\text{M}}_{\text{t}}={\text{M}}_{0}e^{-k\text{t}}$$ (1)

where M_t_ is the litter mass at collection time t, M_0_ is the initial litter mass, and *k* is the decay constant [44]. We measured initial mass and estimated *k* by fitting negative exponential decay curves rather than linear fits of log-transformed data to avoid potential error generated from transforming the data [45]. We opted to use a single-pool decay function because it fit our data well. The single-pool model is also the most commonly used model, making our results comparable to other studies and useful in syntheses. All decay data were analyzed using a mixed effects 3-way ANOVA testing the fixed effects of litter type, photopriming treatment, precipitation treatment, and all possible interactions. Plots were nested within blocks as random effects. Simple linear regression was used to test the relationships between mass remaining and C or N mass remaining, and to generate model fit statistics (*i.e.* AICc; [46]). All data analysis was done using R version 3.4.1 [47]. All data were tested for normality and equal variance *a priori* and deemed suitable for the statistical methods used.

## Results

### Litter type and precipitation drive patterns of decay constants

**Fig 1:**
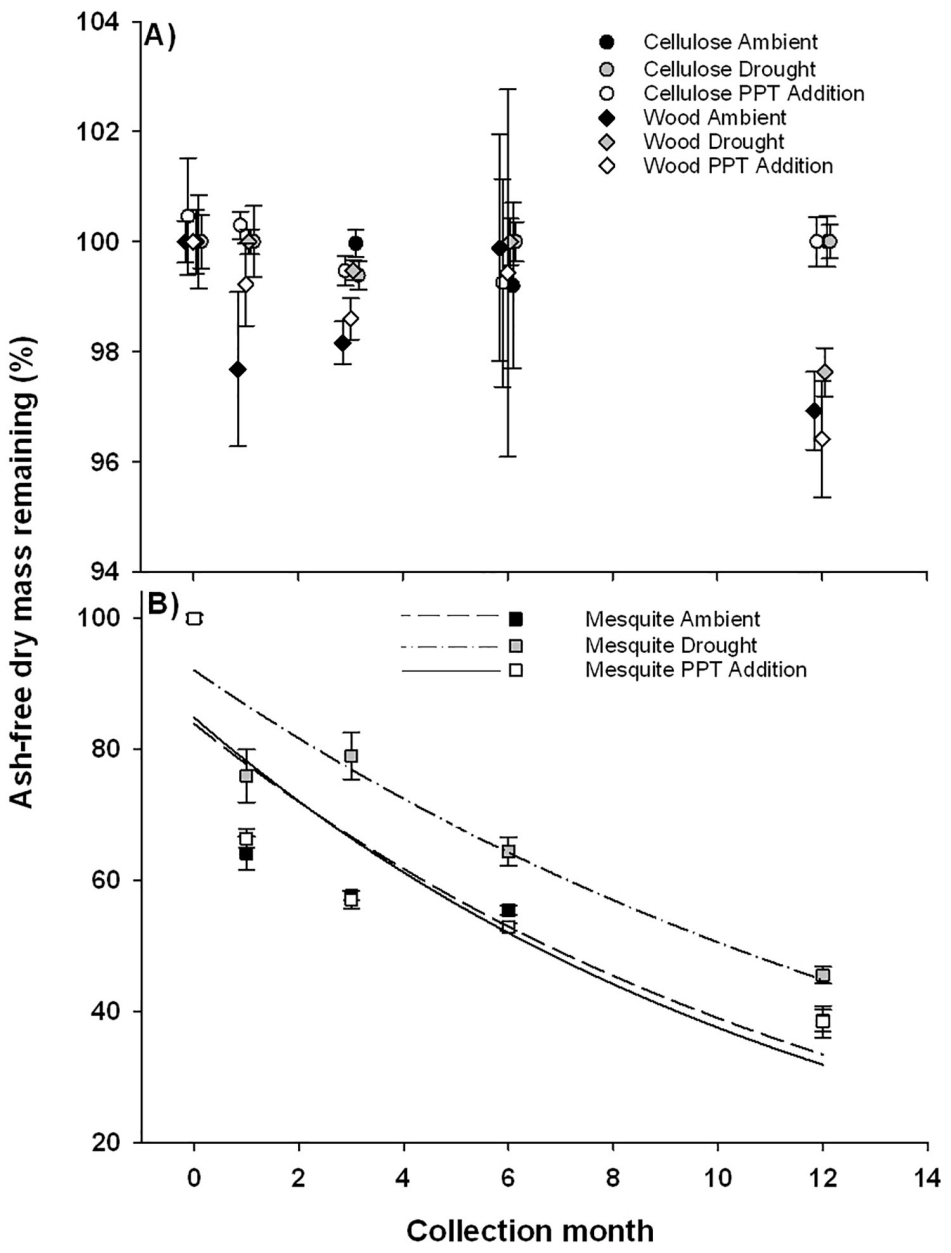
Decay curves of cellulose paper (cell), mesquite (mes), and basswood (wood) under each of the three precipitation treatments including the drought, ambient, and addition manipulations. Symbol shape and fill combinations indicate different litter types. The three exponential decay curves are fitted to mesquite litter as it was the only litter type to show measurable mass loss over the course of the experiment. Mesquite mass loss was reduced under drought (dashed line) relative to ambient (solid line) and precipitation addition (dotted line) treatments. Error bars are standard error.

Photopriming did not affect decay constants (*k* values) for any of the litter type by precipitation combinations (Table 1). Litter decay constants were strongly affected by an interaction between litter type and precipitation (Fig 1; Table 2), such that mesquite litter under ambient (*k* = 1.37 ± 0.04 yr^-1^) and elevated precipitation treatments (*k* = 1.38 ± 0.05 yr^-1^) had the highest decay constants followed by mesquite under drought conditions (*k* = 0.89 ± 0.04 yr^-1^). Both cellulose and basswood showed very low decay constants (*k* = 0.03 ± 0.04 yr^-1^), and were subsequently not affected by precipitation manipulations (Fig 1).

**Table 1:** Summary of mean decay constants (k-values) and associated standard errors (SE; ±) estimated by fitting a single exponential decay function to litter mass loss data. Values are categorized by each litter type by photopriming by precipitation treatment combination used in the study.

Litter	Photopriming	Precipitation	*k*-value	SE
		Drought	-0.03	0.03
	Dark	Ambient	-0.02	0.02
		Addition	-0.05	0.05
		Drought	-0.05	0.07
Cellulose paper	-UVB	Ambient	-0.02	0.04
		Addition	-0.04	0.06
		Drought	-0.01	0.07
	+UVB	Ambient	-0.04	0.03
		Addition	-0.02	0.08
		Drought	0.96	0.15
	Dark	Ambient	1.35	0.19
		Addition	1.39	0.11
		Drought	0.87	0.13
Mesquite	-UVB	Ambient	1.37	0.11
		Addition	1.40	0.09
		Drought	0.84	0.15
	+UVB	Ambient	1.43	0.06
		Addition	1.33	0.18
		Drought	0.01	0.03
	Dark	Ambient	0.02	0.04
		Addition	0.02	0.06
		Drought	0.04	0.04
Basswood	-UVB	Ambient	0.01	0.03
		Addition	0.07	0.09
		Drought	0.04	0.08
	+UVB	Ambient	0.01	0.03
		Addition	0.01	0.05

**Table 2:** Summary of ANOVA model results testing the effects of litter types, photopriming (Pp), precipitation manipulations (PPT), and their interactions on decay constants (*k*values over the course of the experiment). Degrees of freedom (DF) are numerator and denominator respectively, F-Value is the computed test statistic, and P-Value is the asymptotic significance.

Treatments	DF	F-Value	P-Value
Litter type	2, 52	61.55	<0.0001
Photopriming (Pp)	2, 52	0.04	0.96
Precipitation (PPT)	2, 52	0.08	0.93
Litter x Pp	4, 52	0.24	0.91
Litter x PPT	4, 52	6.99	0.0001
Pp x PPT	4, 52	0.08	0.99
Litter x Pp x PPT	8, 52	0.20	0.99

### Precipitation effects on mesquite litter mass remaining

**Fig 2:**
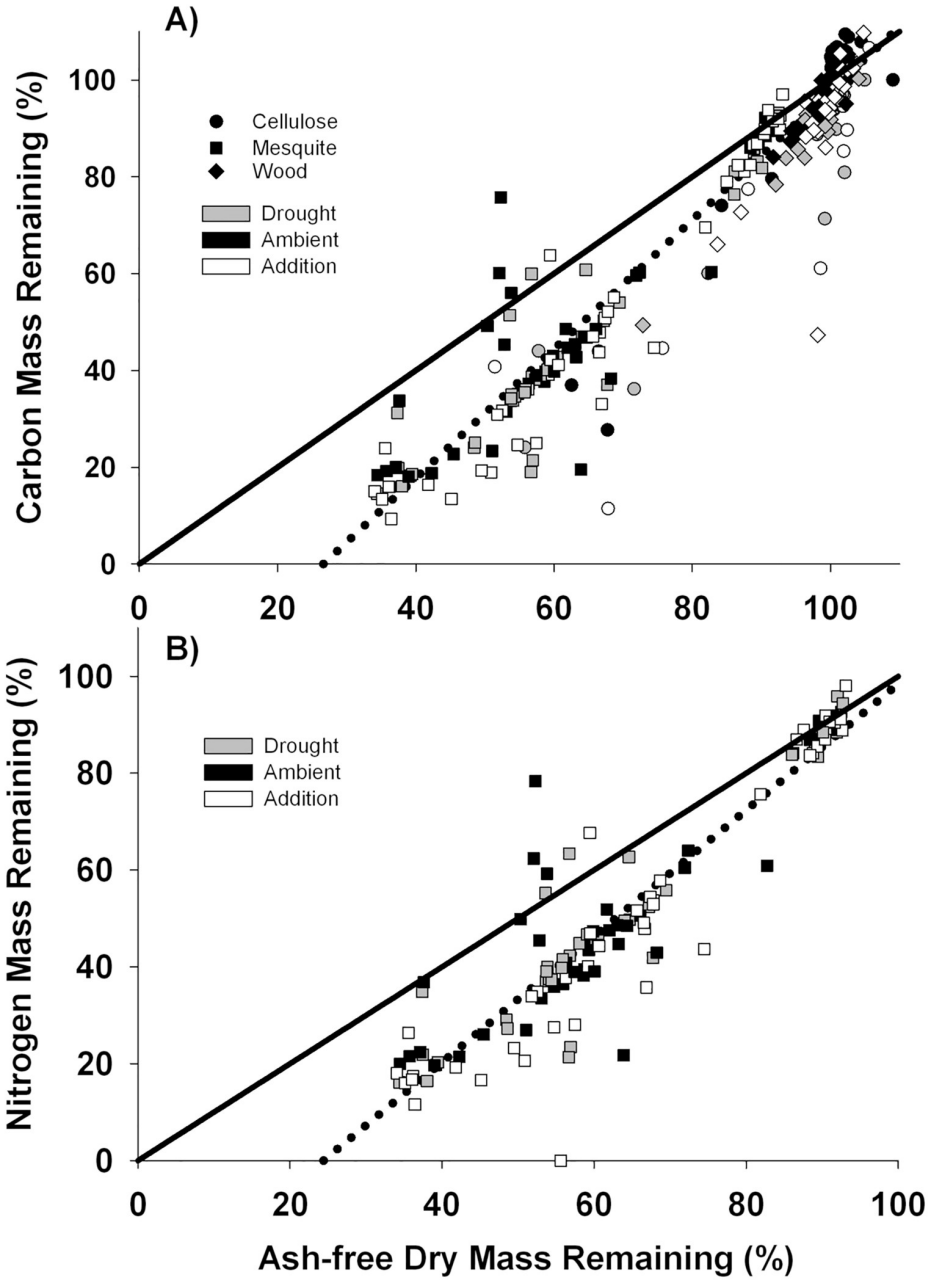
Carbon (A) and nitrogen (B) mass remaining (%) regressed against ash-free dry mass remaining (%) of litter used in this study. Solid lines are 1:1 relationships and the dashed lines are regression lines fitted to the data. Different shape and fill combinations indicate different precipitation and litter type combinations.

Given the limited negligible mass loss of cellulose and basswood, we focused analyses of precipitation effects on mesquite litter. After 12 months, mesquite litter C mass remaining (%) was affected by precipitation treatments (F_2, 283_ = 3.9, P = 0.04) such that C mass remaining was lowest in the precipitation addition treatment (15.9% ± 4.1) followed by the ambient control (20.7% ± 5.2), and the drought treatment (23.7% ± 7.5). Carbon mass remaining (%) of mesquite was positively linearly related to ash-free dry litter mass remaining (%) (Fig 2A; R^2^ = 0.93). Nitrogen mass remaining (%) of mesquite was also affected only by precipitation treatments (F_2,283_ = 3.36, P = 0.05). Nitrogen mass remaining (%) was positively linearly related to ash-free dry litter mass remaining (%) (Fig 2B; R^2^ = 0.85). When examining the effects of precipitation manipulations on mesquite litter mass remaining, ambient control and precipitation addition treatments explained a greater amount of variation in the relationship between mass remaining and C and N mass remaining (Table 3). While variation in C mass remaining was explained by mesquite litter mass remaining under drought treatment, this relationship does not hold for N mass remaining, as variation in N mass remaining was not significantly explained by litter mass remaining in the drought treatment (Table 3).

**Table 3:** Simple linear regression results of the relationship between ash-free dry mass remaining (%) of mesquite litter and mass remaining (%) of carbon and nitrogen of mesquite litter for each of the three precipitation manipulation treatments. Cellulose and basswood were not included in this analysis due to their negligible decomposition.

Mesquite Litter	Carbon Mass Remaining			Nitrogen Mass Remaining		
Precipitation Treatments	R^2^	AICc	P	R^2^	AICc	P
Drought (-)	0.79	335.3	0.002	0.52	369.4	0.2
Ambient	0.91	302.9	<0.001	0.91	295.6	<0.001
Addition (+)	0.93	303.8	<0.001	0.92	297.3	<0.001

## Discussion

Recent studies suggest that abiotic processes including photodegradation from UV radiation [14, 15, 17] may explain part of the discrepancy between modeled and observed patterns in dryland litter decomposition [36, 48]. However, laboratory and field studies indicate interactions between soil-litter mixing and microbial decomposition in dryland ecosystems [11, 13, 29, 49]. Recent conceptual frameworks have sought to integrate these ideas, and have presented a broader understanding of the spatiotemporal dynamics associated with litterfall and abiotic (*e.g.* exposure to UV radiation and soil deposition) and biotic (*e.g.* microbial activity) drivers of decomposition [13, 19, 37], suggesting that photodegradation may play a role in mineralizing standing-dead litter prior to leaf abscission and soil-litter mixing. On the other hand, several other studies suggest that the decay of recalcitrant compounds by photo-oxidation, may, in turn, facilitate microbial decomposition by making the litter more degradable [15, 31, 35, 36]. In this study, we tested a comprehensive suite of decomposition drivers by applying a photopriming treatment designed to mimic exposure of litter to solar radiation prior to abscission, followed by mixing soil and litter in the field to facilitate biotic decomposition. This design allowed for an examination of potential interactions between photopriming and precipitation-mediated drivers of decomposition in drylands. While we did not measure microbial activity directly, we assume that observed mass loss was the result of microbial activity as the buried litterbags negated further photodegradation and restricted access by larger soil organisms. In addition, leaching losses in this dryland ecosystem are assumed to be negligible.

Contrary to our hypothesis that photoprimed litter would have rapid mass loss, particularly in wetter conditions, only mesquite showed distinct mass loss over the year-long study, while cellulose paper and lignin-rich basswood masses did not change. The patterns of mesquite litter C and N mass remaining displayed a similar trend to litter mass remaining. The lack of decay in filter paper and basswood (both undetectable N content) suggests that litter N plays an important role in dryland litter decomposition, and that litter N may be more important than photopriming in regulating decomposition. Additionally, the relationship between litter N and litter mass loss, environmental factors such as precipitation affected litter mass loss rates such that litter mass loss was slower under drought treatment, which aligns with field studies in the northern Chihuahuan Desert [11, 29, 49]. This also suggests that microbial decomposition may be more important in dryland litter decomposition [29], as compared to abiotic degradation including photodegradation of litter, which accounts an estimated 7-23% depending on the study design [17, 18]. Overall, these trends highlights the importance of litter quality (*e.g.* C:N) for litter decomposition even in drylands where environmental conditions strongly regulate decomposition, thus supporting the likelihood of N-dependent biological decomposition.

In our experiment, the extremely low litter N content limited biotic decomposition across the range of precipitation treatments. Moreover, the lack of any measurable decay in filter paper or basswood litter highlights that photopriming did not enable microbial decay by decomposing recalcitrant compounds, as found in other recent studies [15, 35, 36]. In the present study, litter N content positively influences microbial decomposition rates, a trend that has been observed in studies that associate litter quality with decomposability [6, 50]. In addition, lignin/leaf N ratio is one of the best predictors of litter decomposition globally [8], wherein a lower ratio is indicative of greater decomposability.

Soil moisture is positively correlated with the rate of organic matter decomposition until soil oxygen becomes limiting. A laboratory incubation of litter and soil with soil moisture manipulation in dryland litter mass loss and subsequent CO_2_ production demonstrated that relative changes in soil moisture not the absolute amount of soil moisture, have a greater influence on microbial activity [34]. Similarly, a field study in the Chihuahuan Desert found that microbial extracellular enzyme activities in leaf litter samples were positively related to seasonal precipitation patterns [29]. In our study, the conditions that significantly limit microbial decomposition (*i.e.*, lack of litter N or sub-ambient moisture) were critical modulators, while the effects of photopriming were negligible, suggesting that environmental factors influencing microbial decomposition are more important in dryland litter decomposition than abiotic decomposition pathways such as UV radiation regardless of exposure to UV prior to leaf abscission.

Although we observed minimal effects of photopriming in our short-term litter decomposition study, we did observe microbially-mediated decomposition in N-containing mesquite litter in all of our photopriming treatments. In the time span of our study, micro-organisms may preferentially decay more labile carbon compounds, and when the labile compounds are in limited supply the microbes move onto utilizing the recalcitrant compounds. Therefore, photodegradation of litter may be more important in the later stage of litter decomposition, when the labile compounds are depleted for microbial consumption. This may be beyond the time span of our study and future research may consider this point. However, litter decomposition studies using mesquite in the Chihuahuan Desert show that within approximately two years, mesquite litter masses overall are largely depleted to ∼20% of initial weight and will approach 0 percent remaining within 48 months [29, 49]. Moreover, these studies show a high degree of biological decomposition (*e.g.* measures of extracellular enzyme activity) and the development of soil-litter aggregates in litterbags containing N-containing mesquite litter, which is facilitated by precipitation [11, 29, 49]. Additionally, due to the dynamic aeolian and fluvial soil surface processes found in many dryland environments [43], surface litter is rapidly covered by soil, which likely limits exposure to solar radiation [37] having a similar effect as the litter burial method used in this study. Our initial hypothesis that photopriming would enhance biological decomposition was not supported in this short-term litter decomposition study, which used a photopriming treatment that reflected solar radiation exposure in the field (*i.e.* 3-months equivalent exposure). We surmise that this result is due to the likelihood that microorganisms preferentially degrade less recalcitrant organic compounds that are readily available rather than the products of any photo-catalyzed or induced reactions derived from recalcitrant substrates.

## Conclusions

Our results do not support the hypothesis that photopriming enhances decomposition in drylands, nor did it interact synergistically with the precipitation manipulations employed in this study. However, photopriming may affect decomposition processes in the long-term, for instance during litter decomposition after the labile organic compounds are depleted by micro-organisms. Our results support recent studies suggesting that precipitation coupled with environmental factors such as soil mixing and litter burial are critical for controlling decomposition [13, 29, 34, 42, 49]. This suggests that microbial decomposition plays a substantive role in the overall cycling of C and N in drylands. Our results suggest that previous estimates of photodegradation on drylands litter decomposition may be overestimating the role of photodegradation on drylands decomposition as a whole.
